# Self-assembled quantum dot microstructure guided by a microemulsion approach for immunoassays†

**DOI:** 10.1039/c9ra05719f

**Published:** 2019-08-28

**Authors:** Jing Liang, Lei Yu, Xue Li, Jiejing Zhang, Guang Chen, Jianfeng Zhang

**Affiliations:** College of Life Science, Jilin Agricultural University, Key Laboratory of Straw Biology and Utilization, The Ministry of Education Changchun 130118 China zhangjianfeng06@tsinghua.org.cn; Jilin Radio and TV University Changchun 130022 China

## Abstract

Quantum dot microstructures were fabricated through a convenient microemulsion approach in this study. A polymer solution containing a stabilizer was mixed with a quantum dot aqueous solution, to prepare a reversed microemulsion, through shaking. Then, the microemulsion was cast on a solid substrate followed by evaporating steps, resulting in the formation of an ordered porous film. Interestingly, the quantum dot microstructure can be produced at the same time. The immunoassay experiment could be realized by the fluorescent microstructures. The green fluorescence microstructure specifically bound with antigens marked with red color quantum dots, resulting in the enhancement of red fluorescence domains and the decrease of green fluorescence. With the addition of unlabeled antigens, the green fluorescence microstructure was recovered. This strategy implies that the quantum dot pattern has potential on biochip, biosensor, and imaging analysis.

## Abstract 2

Ordered nanocrystal microstructures are conveniently prepared *via* a microemulsion which can further be applied for immunoassays.

## Graphical abstract

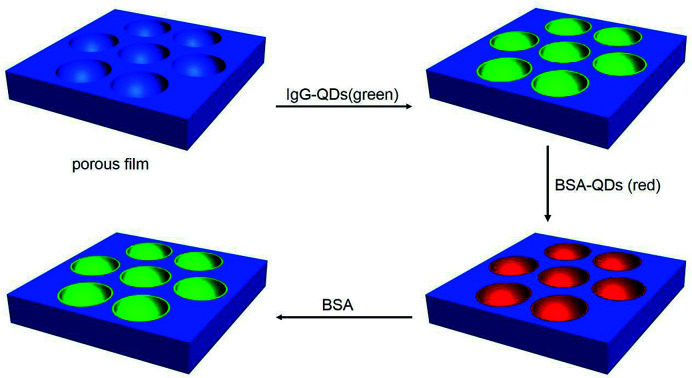

## Introduction

Nanoparticles, which are required to be manipulated into functional materials and devices, could be widely used in the areas of catalysis, optoelectronics, sensors, solar cells, and so on.^1^ Quantum Dots, abbreviated as QDs, as a famous kind of nanoparticles, possessing tunable optical and electrical features, have been widely applied in the field of biosensors, nanoelectronic devices, solar cells, quantum computing, and immunoassays.^2–8^ Thus, methods which can organize QDs into periodically ordered structures are necessary. However, unlike polymers, QDs are not easily processable. In order to take full advantage of the excellent properties of QDs, challenges need to be faced to design ordered structures of QDs. At present, periodical structures usually can be accomplished by photolithography, electron beam lithography, and molecular-beam epitaxy.^9–11^ However, the above mentioned techniques possess the inherent limitations of inconvenience, high cost, and low throughput. Thus, improved approaches that can avoid the disadvantages, are welcome.

Self-assembly methods, have been widely used to prepare nanoparticle surface structures, due to their low-cost characters.^12–14^ As the fast and easy operation features, water-template technique using condensed water droplets as template, has been widely investigated.^15,16^ So far, preparation of QDs pattern structures by using this approach has been reported. CdSe QDs modified by organic molecules, CdS and ZnS QDs were assembled into porous films,^17–19^ as well as silver nanoparticles with CdSe QDs were incorporated into the honeycomb architectures.^20^ Generally, the pattered architectures were prepared by using the materials of polymer and organic soluble QDs. However, the organic molecules decorating QDs which need additional steps to be synthesized and chose appropriate organic ligands, result complicated preparation process of the film, and increase the difficulty to achieve ordered architectures. Usually, the present QDs architectures are in random size, spacing, and periodicity. And, the detail usage of the QDs structures are less mentioned. Thus, to overcome these deficiencies, a convenient method which can operate various kinds of QDs especially water soluble QDs into periodically ordered structures, with detail usage, is necessary.

**Scheme 1:**
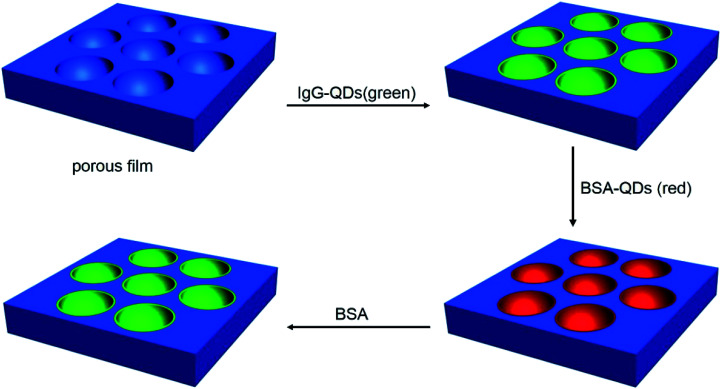
The schematic drawing of assembly of QDs and *in situ* immunoassays.

Immunoassay plays a critical role in clinical, pharmaceutical and environmental chemistry. As a kind of attractive luminescent materials, QDs used in the immunoassay area, especially on the surface for immunoassay which possess visualized advantages, are welcome. Herein, QDs microstructure, has been prepared by microemulsion-assisted method, through pre-adding QDs into water phase following with their incorporation into the cavities on the polymer film. This strategy possess some advantages. The QDs microstructures are formed in one step accompanying with the formation of porous film, saving several complicated procedures for QDs pattern preparation comparing with other methods. Various kinds of water soluble QDs could be employed, which avoid difficult operational features of QDs. In addition, the QDs microstructure are in adjustable dimension and high order, and the optical and biological characters of QDs are maintained. Further, the QDs microstructure can be used to realize immunoassays (Scheme 1). It should be noted that this method for fabrication of QDs microstructures possess convenient, cheap, and easy advantages. It indicates that these microstructures have potentials on microreaction, biochip, biosensor, cell culture, and pattern recognition.

## Material and methods

### Materials

Poly(methyl methacrylate) (PMMA, *M*_w_: 349 kg mol^−1^) and polystyrene (PS, *M*_w_: 349 kg mol^−1^) were obtained from Sigma-Aldrich, USA. Didodecylamine (DDA) and *N*-hydroxysulfo-succinimide (NHS) were from Aladdin, China. PEO_20_-PPO_70_-PEO_20_ (P123) was the product of Anqiushi Luxing Chemical Co. Ltd., China. 1-(3-Dimethylaminopropyl)-3-ethylcarbodiimide hydrochloride (EDC) was purchased from J&K Scientific Ltd, China. BSA was the product of Beijing Biosynthesis Biotechnology Co. Ltd., China. Anti-BSA antibody (IgG) was the products of Invitrogen, USA. The mercaptopropionic acid modified CdTe (QDs) nanoparticles were synthesized according to the literature in this lab.^21^ The BSA-QDs and IgG-QDs were prepared through the procedures from the literature.^22^

### Preparation of QDs microstructures

PMMA (6 mg mL^−1^) and P123 (0.6 mg mL^−1^) were added to a certain volume of dichloromethane to prepare a mixture. The typical procedure for microemulsion preparation was that an aqueous solution of QDs (0.5 mM) with green or red fluorescence was added to the mixture of PMMA and P123, and the volume fraction of water phase was 5%. Then, the above mixture was shaken for 30 s under 25 °C to disperse the water phase in organic phase to achieve a translucent gray microemulsion. Then, 20 μL of the microemulsion was cast onto a glass substrate, under the relative humidity of 30–40% and temperature of 25 °C, to obtain QDs/PMMA porous films. Following the similar procedures, IgG-QDs/PMMA and QDs/PS films was prepared.

### Immunoassays

The general procedure for the immunoassay is that after immersion of the PMMA porous film with green emission IgG-QDs into the BSA-QDs with red emission in pH = 7 PBS buffer solution for 12 h, the fluorescent microstructures were washed with water three time and dried in air. Then, the fluorescent immune-complex microstructure was soaked into the BSA (2 mM) buffer solution for 5 h, followed by washing with water three times and drying in air.

### Measurements

Scanning electron microscopy (SEM) images were collected on a JEOL JSM-6700F field emission scanning electron microscope. Confocal laser scanning microscopic (CLSM) images were obtained on an Olympus fluoview FV1000. Analysis of CLSM data was carried out using the software FV10-ASW.

## Results and discussion

### Preparation and structural characterization of porous film

**Fig. 1:**
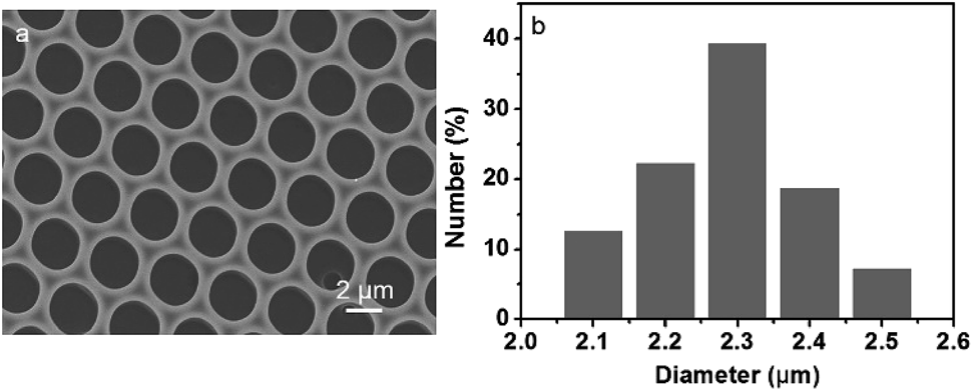
SEM images viewed from the (a) top surface and (b) histogram referring to the size distribution of cavities of the porous film.

We recently developed an effective method to prepare ordered porous surface structures on polymer film using emulsion.^23–25^ Water-phase additives automatically assembled into the cavities, which are suitable for further application in the limited units. This approach not only maintains the advantages of breath figure, but also saves the extra procedures for preparation and selection of appropriate QDs. In this strategy, the QDs microstructure can be prepared simultaneously with the formation of porous film. Typically, the microemulsion was prepared through a slight shaking of the mixture which was consisted of PMMA and P123 dichloromethane solution and aqueous solution of CdTe QDs. After that, the microemulsion was obtained. Then, the microemulsion was cast on a glass slide followed by solvent evaporation, the QDs microstructures was obtained under the certain temperature and humidity. The obtained microstructures display bright iridescent colors when viewed along the reflection light, revealing a periodic refractive index variation regarding the film thickness. The surface morphology of film was characterized through SEM analysis. The SEM image shows that the spherical cavities spread uniformly in a well-ordered array throughout the film, as displayed in Fig. 1a. The histogram (Fig. 1b) displays an even cavity size distribution within 2.1–2.5 μm in diameter.

### The characterization of QDs microstructures

**Fig. 2:**
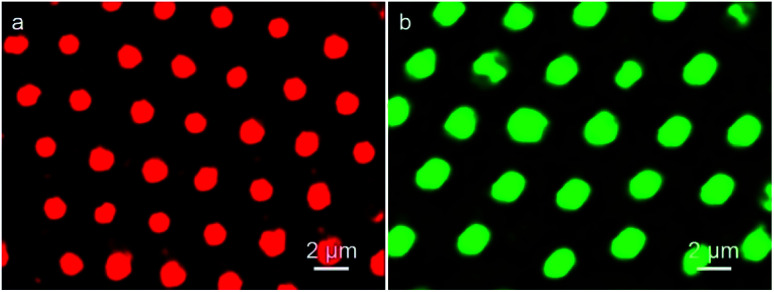
CLSM images of PMMA porous film with (a) red and (b) green fluorescent QDs.

In this strategy, to realize the formation of QDs microstructure in one step, QDs act as the additives which previously incorporated into the water phase. After that, the QDs can automatically assemble into the cavities accompanying with the formation of porous structure. As seen in Fig. 2a, discrete fluorescent circular domains from red fluorescent emission QDs, demonstrates the preparation of QDs microstructures. Similarly, when the green fluorescent QDs was adopted, discrete green fluorescent circular arrays were achieved. Due to the pre-fixing of QDs in the water phase, when the organic solvent and water evaporate completely, the imprint of QDs containing aqueous droplets that play as the template builds the cavities, so that the QDs remain in the cavities and PMMA locates at the exterior. Thus, the red and green color QDs microstructures were prepared in one step. However, when the materials for the preparation of porous structures was replaced by PS and DDA, discrete fluorescent ring structures were obtained due to the ionic interaction between QDs and DDA (Fig. S1†). The reason is that weak interaction between the stabilizer of P123 and QDs leads to accumulation of QDs additives in the bottom of the cavities after the solidification of the films, but the strong electrostatic force between the stabilizer of DDA and QDs gives rise to the deposition of QDs additives in almost the whole surface of the cavities. The one-step prepared QDs microstructures reveal the efficiency of this strategy for the QDs microstructures fabrication, simplifying experimental process and ensuring the regularity of the microstructures. In the microemulsion method, QDs behave only as the additives, which implies that almost all kind of water-soluble but organic-insoluble QDs are available to form this type of microstructures.

### Immunoassays based on QDs microstructure

Immunoassay are widely applied on clinic and pharmaceutics. The famous one is the assay based on the principle of antigen–antibody binding. The attractive fluorescent characteristics of QDs suggest that they could be advisable materials for immune-molecule labeling, comparing with traditional organic dyes. Due to their rather wide excitation band and quite narrow emission spectra, the wavelengths of the QDs could be modulated over a wide range according to the alternation of QDs' dimension. Therefore, the immunoassay which bases on the different imaging color due to the different sizes of the same kinds of QDs materials, is feasible. The immunoassay are usually realized in the solution, but the analysis transferred to interface and surface could be more intuitive.

**Fig. 3:**
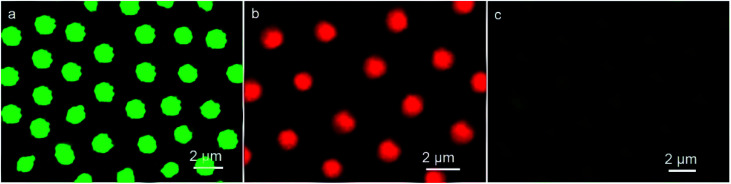
CLSM images of IgG-QDs microstructure before (a) and after encountering the immersion in BSA-QDs solution observed in (b) red and (c) green fluorescent modes.

In this strategy, immunoassay was carried out using the well-known sandwich assay. Typically, the green fluorescent IgG-QDs was prepared, then was assembled into the cavities. Seen in Fig. 3a, comparing with CLSM result of uncoupled CdTe QDs pattern, similar discrete fluorescent circular domains of IgG-QDs on the film are achieved. When the IgG-QDs microstructure is immersed into red fluorescent BSA-QDs buffer solution, the results display that the strong red fluorescent microstructure dating from BSA-QDs appear at the position where the green fluorescent IgG-QDs locate (Fig. 3b), but the previous green fluorescent microstructure becomes weaken (Fig. 3c). When IgG-QDs with green luminescence is combined with red fluorescent BSA-QD, the IgG-QDs/BSA-QDs immune-complex was formed. This immune-complex makes the two QDs close enough, following with the appearance of Förster resonance energy transfer (FRET), which is a nonradiative process whereby an excited state donor transfers energy to a proximal ground state acceptor through long-range dipole–dipole interactions. The acceptor must absorb energy at the emission wavelength of the donor, but does not need to eliminate its own energy.^26,27^ Thus, the energy of the excitonic state in the green fluorescent QDs is transferred to the similar state of the QDs with red emission who are in lower exciton energy. As the strong overlap of the emission and absorption spectra of the used QDs, the efficiency of this FRET process is quite high.

**Fig. 4:**
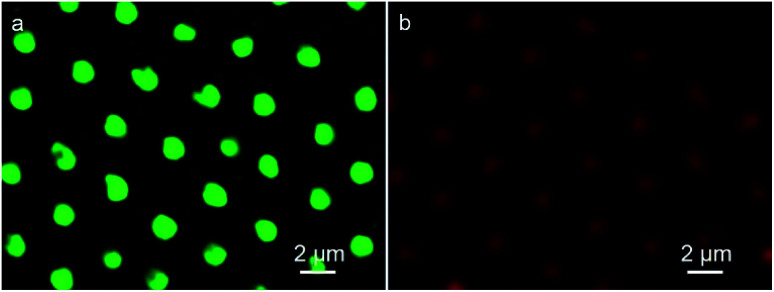
CLSM images of microstructure with immunocomplex after immersion in the unlabeled BSA solution observed in (a) green and (b) red fluorescent modes.

Importantly, when the unlabeled BSA was added, the green fluorescent microstructures dating from IgG-QDs was recovered, followed with the decrease of red fluorescence of BSA-QDs (Fig. 4a and b). The addition of unlabeled BSA destroyed the immune-complex, competitively binding to IgG-QDs and inhibiting the FRET process. Hence, the green fluorescent microstructure was obtained, accompanying with the decrease of the red fluorescent microstructure. Thus, the strategy employs an alternative route to realize a quite visual imaging immunoassays. It can be envisioned that other immunoassays are available in this system, such as human albumin with their antibody.

## Conclusion

In conclusion, the self-assembled QDs microstructures have been prepared by the microemulsion strategy, which has the ability to incorporate QDs into the cavities in one step. The preparation of QDs microstructures are much more convenient and cheap comparing with other methods. All kinds of water soluble QDs could be used to construct various QDs microstructures and the QDs microstructure are in adjustable dimension and high order, indicating the universality of this strategy. In addition, the optical and biological characters of QDs are maintained, giving rise to realization of immunoassay for the QDs microstructures. It can be envisioned that these microstructures have potentials on biosensor, biomedicine, and biochips.

## Conflicts of interest

There are no conflicts to declare.

## Supplementary Material

RA-009-C9RA05719F-s001
