# Supplementary material for: Self-assembled quantum dot microstructure guided by a microemulsion approach for immunoassays
Source: RSC Adv. 2019 Aug 28;9(46):26838–42. doi: 10.1039/c9ra05719f (PMC9070542; doi:10.1039/c9ra05719f)
Supplement: RA-009-C9RA05719F-s001 [file RA-009-C9RA05719F-s001.pdf]

Electronic Supplementary Material (ESI) for RSC Advances.
This journal is © The Royal Society of Chemistry 2019

Supplementary Information

Self-assembled Quantum Dots Microstructure Guided by Microemulsion Approach for Immunoassays

Jing Liang,[a] Lei Yu,[b] Xue Li,[a] Jiejing Zhang,[a] Guang Chen,[a] Jianfeng Zhang*[a]

[a] College of life science, Jilin Agricultural University, Key Laboratory of Straw Biology and Utilization, the Ministry of Education, Changchun 130118, China

[b] Jilin Radion and TV University, Changchun 130022, China

E-mail: zhangjianfeng06@tsinghua.org.cn

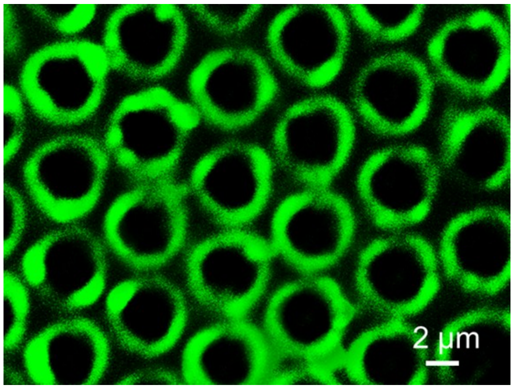

Fig. S1 CLSM images of PS porous film with green fluorescent QDs.
